# Complete Genome Sequence of *Agrobacterium* sp. Strain 33MFTa1.1, Isolated from *Thlaspi arvense* Roots

**DOI:** 10.1128/MRA.00432-19

**Published:** 2019-09-12

**Authors:** Sasha Langley, Thomas Eng, Kenneth H. Wan, Robin A. Herbert, Andrew P. Klein, Yasuo Yoshikuni, Susannah G. Tringe, James B. Brown, Susan E. Celniker, Jenny C. Mortimer, Aindrila Mukhopadhyay

**Affiliations:** a BioSciences Area, Lawrence Berkeley National Laboratory, Berkeley, California, USA; b Joint BioEnergy Institute, Emeryville, California, USA; c Department of Biology, University of North Carolina at Chapel Hill, Chapel Hill, North Carolina, USA; d Howard Hughes Medical Institute, University of North Carolina at Chapel Hill, Chapel Hill, North Carolina, USA; e U.S. DOE Joint Genome Institute, Walnut Creek, California, USA; f Centre for Computational Biology, Biosciences, University of Birmingham, Birmingham, United Kingdom; g Department of Statistics, University of California, Berkeley, California, USA; Loyola University Chicago

## Abstract

*Agrobacterium* sp. strain 33MFTa1.1 was isolated for functional host-microbe interaction studies from the *Thlaspi arvense* root-associated microbiome. The complete genome is comprised of a circular chromosome of 2,771,937 bp, a linear chromosome of 2,068,443 bp, and a plasmid of 496,948 bp, with G+C contents of 59%, 59%, and 58%, respectively.

## ANNOUNCEMENT

Agrobacterium is a diverse genus of soil-dwelling bacteria in the alphaproteobacterial family Rhizobiaceae. Many *Agrobacterium* species cause plant diseases, including Agrobacterium tumefaciens (crown gall disease), Agrobacterium rhizogenes (hairy root disease), and Agrobacterium vitis (lesions and tumors on grape vines). First described in 1897 (1), *Agrobacterium* has been widely studied, largely because of its ability to transform plant cells with its DNA (which is known as transfer DNA [T-DNA]). As a result, A. tumefaciens has become the workhorse of plant genetic engineering (2–4). Other strains of *Agrobacterium* are commensal inhabitants of plant tissue. For example, *Agrobacterium* sp. strain 33MFTa1.1 was isolated from the root endophytic compartment of Thlaspi arvense, a close relative of the model plant Arabidopsis thaliana (5), and recolonizes gnotobiotic A. thaliana plants without producing disease symptoms (6, 7). This report describes the complete genome sequence of *Agrobacterium* sp. 33MFTa1.1 and will facilitate plant-microbe interaction studies.

*Agrobacterium* sp. 33MFTa1.1 (NCBI taxon identifier [ID] 1279031) was obtained from Jeff Dangl. A previously published (5) draft shotgun assembly of this strain consists of 15 contigs, and we posited that long-read sequencing techniques would enable assembly at the chromosome level. Bacteria were streaked onto LB plates, single colonies were amplified, and an aliquot was used for 16S V1 and V4 PCR (8) and sequence identification (reviewed in reference 9). DNA was isolated (10), and whole-genome sequencing was performed at Lawrence Berkeley National Laboratory (LBNL) using a combination of Oxford Nanopore long-read sequencing on the MinION Mk1B (11) and Illumina paired-end 300-bp read sequencing for quality (12). Oxford Nanopore sequencing libraries were constructed from 5 to 10 μg DNA using the Oxford Nanopore 1D native barcoding genomic DNA protocol (version NBE_9006_v103_revO_21Dec2016) and sequenced on three FLO-MIN107 R9 version flow cells. Oxford Nanopore data were demultiplexed with Porechop (13). Sequencing yielded 61,147 reads with a length of ≥2,000 bp and a filtered mean read length of 6,862 bp, totaling 419,565,480 bp (∼79-fold coverage). The Illumina sequencing library was constructed from 1.5 μg DNA. The DNA was fragmented using a Diagenode Bioruptor, and libraries were constructed using the NEBNext Ultra DNA library prep kit for Illumina. Sequencing yielded 2,529,890 paired-end reads, which were trimmed using Trimmomatic (14), resulting in a filtered mean read length of 270 bp and totaling 631,387,669 bp (∼128-fold coverage). Nanopore and Illumina sequencing data were used as inputs for a *de novo* hybrid assembly constructed using Unicycler version 0.4.1 with the “bold” option (15, 16). The assembly produced 3 contigs, a single circular chromosome, a single linear chromosome, and a plasmid. Annotations of protein-encoding open reading frames and noncoding RNAs (ncRNAs) were predicted with the NCBI Prokaryotic Genome Annotation Pipeline (17).

The circular chromosome annotation predicts 2,654 protein-coding genes, 63 pseudogenes, 2 rRNA operons, and 40 tRNAs, with canonical anticodon triplets that base pair with codons for amino acids. It also encodes the telomerase A (*telA*) gene that encodes the protein required to generate the covalently closed hairpin loops at the ends of linear chromosomes (3). The linear chromosome annotation predicts 1,800 protein-coding genes, 69 pseudogenes, 2 rRNA operons, and 14 tRNAs. In addition, the genome includes a single 496,948-bp plasmid, p_JBx_073812, which contains candidate genes for plasmid replication initiation proteins (*repA*, *repB*, and *repC*) and for conjugative transfer (*traA, traB, traC, traD, traF, traG, traH*, and *traM*). It also carries genes for arsenic resistance (*arsH* and *ACR3*) and arsenate metabolism (two copies of the arsenate reductase gene *arsC*). A comparison of the new assembly with the previous 15-contig assembly (NCBI taxon ID 1279031) using the Joint Genome Institute (JGI) microbial species identifier (MiSI) genome-wide average nucleotide identity, alignment fraction (gANI, AF) calculator (https://ani.jgi-psf.org/html/calc.php) reveals high similarity, as expected, with gANI values of 100 and AF values of 0.99 (previous assembly→new assembly) and 0.98 (new assembly→previous assembly) (18).

To identify gene clusters of interest for further research, we analyzed the genome with the antibiotics and Secondary Metabolite Analysis SHell (antiSMASH) version 4.2.0 (19) tool. A total of 41 clusters and putative clusters were identified. These included a type I polyketide synthase cluster, a terpene cluster, and a nonribosomal peptide synthetase cluster. Of the remainder, 5 were putative fatty acid clusters, 5 were putative saccharide clusters, and 28 were putative clusters of unknown type, as identified by the ClusterFinder algorithm (19).

### Data availability.

The complete circular and linear chromosomes and plasmid sequences described here are deposited in GenBank under the accession numbers CP036358 (circular chromosome), CP036359 (linear chromosome), and CP036360 (plasmid). The SRA accession number is PRJNA523206.
